# Development of consensus-based aims, contents, intended learning outcomes, teaching, and evaluation methods for a history of medicine and pharmacy course for medical and pharmacy students in the Arab world: a Delphi study

**DOI:** 10.1186/s12909-021-02820-7

**Published:** 2021-07-16

**Authors:** Ramzi Shawahna

**Affiliations:** 1 Department of Physiology, Pharmacology and Toxicology, Faculty of Medicine and Health Sciences, An-Najah National University, New Campus, Building: 19, Office: 1340, P.O. Box 7, Nablus, Palestine; 2 An-Najah BioSciences Unit, Centre for Poisons Control, Chemical and Biological Analyses, An-Najah National University, Nablus, Palestine

**Keywords:** Education, History of medicine, Pharmacy, Consensus, Delphi technique

## Abstract

**Background:**

History courses are “required” elements among the didactic elements of the medical and pharmacy curricula in many schools around the world. The aim of this study was to develop consensus-based aims, contents, intended learning outcomes, teaching, and evaluation methods of a history of medicine and pharmacy course for medical and pharmacy students in the Arab World.

**Methods:**

A systematic search of PubMed, ScienceDirect, SpringerLink, Scopus, and Google Scholar was conducted to identify course aims, contents, intended learning outcomes from the literature. The search was supplemented by semi-structured in-depth interviews with 5 educators/academicians, 3 pharmacists, and 3 physicians. The Delphi technique was used among panelists (10 educators/academicians, 4 physicians, and 4 pharmacists) to develop consensus-based course aims, contents, intended learning outcomes, teaching, and evaluation methods.

**Results:**

The vast majority of the panelists agreed on the 10 items (agreement ≥88.9%) on the importance of teaching history to medical and pharmacy students. Consensus-based aims (*n* = 4) and intended learning outcomes (*n* = 13) were developed in the 1st and 2nd iterative Delphi rounds. The panelists suggested that 16 dedicated meeting hours (1 credit hour) would be required to cover the course. Bloom’s verbs were used to target the lower and higher orders of the cognitive domain. The course could be taught through face-to-face lectures, provision of reading materials, video documentaries, case studies, group discussions and debates. Multiple-choice questions, written reflections, portfolios, group projects, and engagement in discussions and debates might be used to evaluate performance of students.

**Conclusion:**

Consensus-based course of history of medicine and pharmacy course was developed for medical and pharmacy students in the Arab World. Well-designed course aims, contents, intended learning outcomes, teaching, and evaluation methods are more likely to meet the accreditation requirements and might improve performance of medical and pharmacy students. Future studies are still needed to investigate if such consensus-based courses can improve performance of the students.

**Supplementary Information:**

The online version contains supplementary material available at 10.1186/s12909-021-02820-7.

## Background

History courses are “required” elements among the didactic elements of the medical and pharmacy curricula in many schools around the world [1]. In the US, history of pharmacy was listed among the required elements in “Standards 2016” report released by the Accreditation Council for Pharmacy Education [1, 2]. Topics suggested by the Accreditation Council for Pharmacy Education to be taught to pharmacy students included evolution of the pharmacy profession, major turning points within the profession, major milestones, discoveries and achievements, and individuals who made significant contributions to the evolution of the profession [2]. Historically, the American Institute of the History of Pharmacy provided recommendations and support to assist schools meet the required curricular standards in teaching history of pharmacy and medicine [3, 4].

Previous studies have shown that many medical and pharmacy schools taught history either as standalone required or elective courses or designated few hours of instruction time to teach few history topics that were inserted into some orientation courses [1, 3, 5]. Older studies showed that of the then 70 schools offering pharmacy degrees in the US, about half offered a course of history to pharmacy students [1]. Of those, 28 schools offered required courses entirely devoted to history. With the advancements of knowledge, medical and pharmacy schools became increasingly pressurized to introduce more courses related to therapeutics and clinical sciences. As a result, there was a decline in offering courses devoted to history in the standard medical and pharmacy curricula in many schools around the world [6, 7].

Baker et al. investigated how pharmacy schools in the US met the standards of teaching history, assessed whether pharmacy schools needed to expand teaching of history, inquired on what pedagogical assistance was needed to expand teaching of history, and determined if elective courses in history were offered to pharmacy students [1]. The study showed that requirements of the Accreditation Council for Pharmacy Education were met by 86% of the schools included in the study. The majority of the schools (72%) devoted 1–5 h of instruction time to meet the accreditation requirements. The study also highlighted that the majority (68%) of the schools did not use supporting literature in teaching history and lacked standard textbooks. The vast majority of the schools (91%) required pedagogical assistance like packaged courses, syllabi, assignments, and assessment tools.

In Palestine, history of medicine and pharmacy is an elective course taught to medicine and pharmacy students. Since its inception, the course was taught differently depending on the choices of the course instructors. Because accreditation of medical and pharmacy education has become a top priority for medical and pharmacy schools [8], the aims, contents, and intended learning outcomes of the courses need to be standardized. Formal consensus techniques were extensively used to develop consensus-based course contents. Since its inception, the Delphi technique has emerged as one of the most commonly used formal consensus method in healthcare. The aim of this study was to develop consensus-based aims, contents, intended learning outcomes, teaching, and evaluation methods of a history of medicine and pharmacy course for medical and pharmacy students in the Arab World.

## Methods

### Study design

**Fig. 1:**
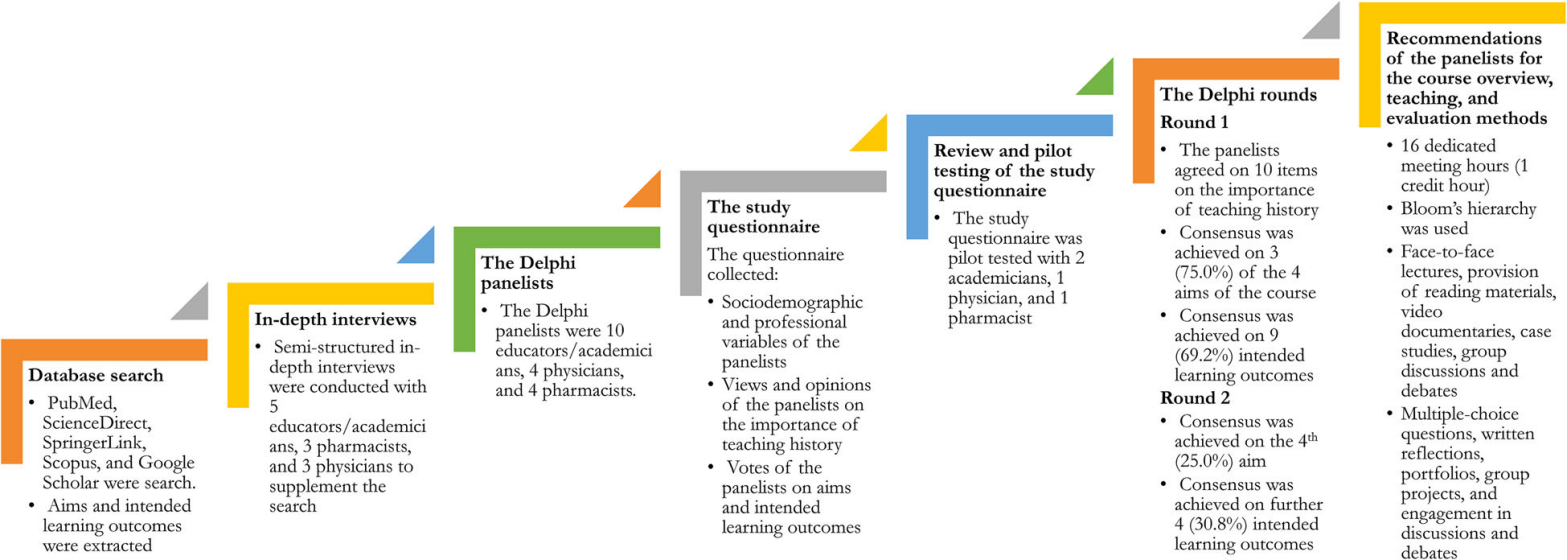
Flowchart of the different stages of the study

This study was conducted using the Delphi technique as a formal consensus technique. Therefore, this manuscript adheres to the Conducting and REporting of DElphi Studies (CREDES) checklist [9]. Adherence to the CREDES checklist is shown in Supplementary Table S1. The different stages of the study are shown in Fig. 1.

### Planning and process

#### Search of databases

The databases: PubMed, ScienceDirect, SpringerLink, Scopus, and Google Scholar were used to search for objectives and intended learning outcomes of history of medicine and pharmacy courses. The text words [TW] and Medical Subject Heading (MeSH) terms relevant to “Education”, “Curriculum”, “Teaching”, “Learning”, AND “History” AND “Medicine” OR “Pharmacy” were combined. The retrieved articles were reviewed to identify and extract course objectives and intended learning outcomes. The search methodology was informed by previous studies [10, 11].

#### In-depth interviews

Semi-structured in-depth qualitative interviews were conducted with educators/academicians and professionals (5 educators/academicians, 3 pharmacists, and 3 physicians) to explore their views and opinions on the objectives and intended learning outcomes of the history of medicine and pharmacy course. The aim of these interviews was to obtain additional objectives and intended learning outcomes that might not be provided in the retrieved articles [12–16]. The interviews were transcribed verbatim and the transcripts were analyzed thematically [12].

#### Selection of the panelists for the Delphi technique

In this study, the panelists for the iterative Delphi rounds were selected using a purposive sampling technique [12–16]. Personal contacts in the field were used to identify, invite, and recruit the panelists who were educators/academicians (*n* = 10), physicians (*n* = 4), and pharmacists (*n* = 4). The educators/academicians, pharmacists, and physicians who participated in the in-depth interviews were also included in the panel. The panel size was informed by previous studies in the domain. It is noteworthy mentioning that there is no consensus on the ideal size of the panel in the Delphi technique. However, previous studies used panels in the sizes from 10 to 1000. It has been argued that the process of panelists selection is one of the most sensitive steps in the Delphi technique [12–17]. Prior knowledge of the topic being investigated is often a prerequisite for selection into a Delphi panel. In this study, the educators/academicians were selected based on their prior knowledge and practical experience in teaching history of medicine and pharmacy. The physicians and pharmacists were selected as professionals who had interest in history of medicine and pharmacy and possessed practical experience in the fields of medicine and pharmacy. The panelists were diversified in terms of their gender, age groups, degrees, type of employer, and length of practical experience. The aim and design of the study was explained to the panelists before their written informed consent to participate was obtained.

#### The study questionnaire

Course objectives and intended learning outcomes that were collected from the literature search and from the qualitative interviews were included into a questionnaire [10, 12, 17]. The questionnaire was composed of 3 sections. The 1st section collected the sociodemographic and professional variables of the panelists like gender, age, academic degrees, profession, type of employer, and length of practical experience. The 2nd section collected views and opinions of the panelists on 10 items relevant to the importance of teaching history of medicine and pharmacy to medical and pharmacy students. On each item, the panelists had to express their views and opinions by responding by either disagree/neutral/agree. The 3rd section collected votes of the panelists on 4 items relevant to the general aims of the course and 13 items relevant to the intended learning outcomes of the course. The panelists had to express their views and opinions on a Likert scale of 1–9.

#### Piloting and review of the questionnaire

Prior to use in this study, the questionnaire was pilot tested with 2 academicians, 1 physician, and 1 pharmacist for comprehensibility and clarity. Of the participants in the pilot testing, 1 academician and 1 pharmacist also participated in the panel. Based on the feedback of the pilot testing, items were rephrased for clarity.

#### The first Delphi round

In the 1st Delphi round, the questionnaire was distributed to the panelists and each panel responded to the questionnaire by filling their sociodemographic and professional variables in the 1st section, expressing their views and opinions on the 10 statements in the 2nd section, and voting on each item in the 3rd section.

#### Analysis of the votes and definition of consensus

In this study, the definition of consensus was informed by previous studies in the domain [12–17]: 1) when the median vote of the panelists was within the range 7–9 and the interquartile range was < 2, consensus was said to have been achieved and the aim or intended learning outcome was included in the final list of aims and intended learning outcomes of the course, 2) when the median vote the panelists was within the range 1–3 and the interquartile range was ≤2, consensus was said to have been achieved and the aim or intended learning outcome was excluded from the final list of aims and intended learning outcomes of the course, and 3) when the median score was within the range 4–6 and/or the IQR was > 2, the item was considered as equivocal. In this study, it was decided a priori that all equivocal items would be subjected to subsequent iterative Delphi round.

#### The second Delphi round

All equivocal items were included in a revised questionnaire and subjected to a 2nd round Delphi. For each item, the panelists were provided with a reminder of their own vote in the 1st Delphi round, the median vote of the other panelists, and the IQR. The panelists were asked if they wished to reconsider their votes after considering the votes of the other panelists or they wished to re-confirm their prior votes. Votes of the 2nd Delphi round were analyzed using the same definitions used in the 1st Delphi round.

#### Proposed course overview, teaching, and evaluation methods

Based on the votes of the panelists, the principal investigator proposed the course overview, teaching, and evaluation methods. The proposed plan was sent to the panelists for review and comments [17]. The panelists were asked to submit their detailed review and comments on the proposed the course overview, teaching, and evaluation methods. The panelists were also encouraged to include their suggestions, views, and opinions. Comments and suggestions of the panelists were analyzed qualitatively. Summaries of the suggestions, views, and opinions with representative quotations were returned to all panelists for a final review. The panelists were requested to comment on the suggestions, views, and opinions either by agreement or rebuttal.

#### Ethics approval and consent to participate

This study was conducted in compliance with the ethical standards in the Declaration of Helsinki and those followed at An-Najah National University. The study was part of the project to improve education of medical and other healthcare professions which received approval from the Institutional Review Board of An-Najah National University. The panelists provided written informed consent before they took part in the study.

## Results

### The panelists

In this study, a total of 18 panelists took part in the voting process. Of those, 8 (44.4%) were female, 11 (61.1%) were 45 years and older, 10 (55.6%) were educators/academicians, 10 (55.6%) had a PhD, 10 (55.6%) were employed by an academic institution, and 12 (66.7%) had 10 or more years of practical experience. The panelists in this study were from the Arab region. The detailed variables of the panelists are shown in Table 1.

**Table 1:** Sociodemographic and professional variables of the panelists (*n = 18*)

Variable	n	%
**Gender**		
Male	10	55.6
Female	8	44.4
**Age group**		
< 45	7	38.9
≥ 45	11	61.1
**Country of origin**		
Palestine	9	50.0
Jordan	2	11.1
Egypt	2	11.1
Morocco	1	5.6
Syria	2	11.1
Tunisia	1	5.6
United Arab Emirates	1	5.6
**Profession**		
Educator/academician	10	55.6
Physician	4	22.2
Pharmacist	4	22.2
**Academic degree**		
BSc/Pharm.D	2	11.1
MSc	2	11.1
MD	4	22.2
PhD	10	55.6
**Employer**		
Academic institution	10	55.6
Hospital/clinic	4	22.2
Pharmacy	4	22.2
**Length of practical experience (years)**		
< 10	6	33.3
≥ 10	12	66.7

*BSc* Bachelor of Science, *CAM* complementary and alternative medicine, *MD* Doctor of Medicine, *MSc* Master of Science, *Pharm.D* Doctor of Pharmacy, *PhD* Doctor of Philosophy

### Views and opinions of the panelists on the importance of teaching history of medicine and pharmacy courses to medical and pharmacy students

The panelists expressed their views and opinions on the importance of teaching history of medicine and pharmacy to medical and pharmacy students. In general, the vast majority of the panelists tended to agree all items (agreement ≥88.9%). The panelists did not disagree with any of the items. Detailed views and opinions of the panelists on the importance of teaching history of medicine and pharmacy courses are shown in Table 2.

**Table 2:** Views and opinions of the panelists on the importance of teaching history of medicine and pharmacy to medical and pharmacy students

		Disagree		Neutral		Agree	
#	Importance	n	%	n	%	n	%
1	Knowledge of history provides insights into what and how to investigate.	0	0.0	1	5.6	17	94.4
2	Knowledge of history is a good antidote against errors, egotism, and despondency.	0	0.0	1	5.6	17	94.4
3	Teaching history of medicine and pharmacy enhances knowledge, gratifies curiosity, broadens views, and strengthens wisdom and judgment.	0	0.0	0	0.0	18	100.0
4	Courses of history of medicine and pharmacy permits highlighting neglected and overlooked discoveries and milestones.	0	0.0	0	0.0	18	100.0
5	Courses of history of medicine and pharmacy provides a stimulus of high ideals students need to have before them.	0	0.0	1	5.6	17	94.4
6	Courses of history of medicine and pharmacy teaches students to cherish the best traditions.	0	0.0	1	5.6	17	94.4
7	Courses of history of medicine and pharmacy helps students tight the bond with their future profession.	0	0.0	1	5.6	17	94.4
8	Knowledge of history allows fulfilling the duty of cherishing the memories, virtues, and/or accomplishments of those who brought benefits to the world as no others have.	0	0.0	0	0.0	18	100.0
9	Courses of history of medicine and pharmacy teaches students to venerate what is good.	0	0.0	2	11.1	16	88.9
10	Knowledge of history enables students feel proud of those remembered for their memories, virtues, and/or accomplishments.	0	0.0	1	5.6	17	94.4

### Consensus-based general aims of the course

In the 1st Delphi round, consensus was achieved on 3 (75.0%) of the 4 aims of the course. In the 2nd Delphi round, consensus was achieved on the 4th (25.0%) aim. Details of the votes of the panelists on each aim in both Delphi rounds are shown in Table 3.

**Table 3:** Consensus-based general aims of the course

		Round 1				Round 2			
#	Aim	Median	Q3	Q1	IQR	Median	Q3	Q1	IQR
1	Providing students with a thorough knowledge and understanding of the history of medicine and pharmacy.	8.0	8.8	7.0	1.8	na	na	na	na
2	Enabling students to recognize the achievements of different individuals and civilizations in the fields of medicine and pharmacy.	8.0	8.8	7.3	1.5	na	na	na	na
3	Enabling students to understand the principles, fundamentals, beliefs, and theories behind disease and treatment in different civilizations.	5.0	7.0	4.0	3.0	7.0	9.0	7.0	2.0
4	Providing students with an understanding of the most important turning points and achievements in the history of medicine and pharmacy.	7.0	8.0	7.0	1.0	na	na	na	na

*na* not applicable, *Q1* 1st quartile, *Q3* 3rd quartile, *IQR* interquartile range

### Consensus-based intended learning outcomes

In the 1st Delphi round, consensus was achieved on 9 (69.2%) of the 13 intended learning outcomes. In the 2nd Delphi round, consensus was achieved on further 4 (30.8%) intended learning outcomes. Details of the votes of the panelists on each intended learning outcome in both Delphi rounds are shown in Table 4.

**Table 4:** Consensus-based intended learning outcomes of the course

		Round 1				Round 2			
#	Outcome	Median	Q3	Q1	IQR	Median	Q3	Q1	IQR
1	Describe the evolution of understanding, diagnosis, and treatment of diseases across successive civilizations.	5.5	7.0	5.0	2.0	7.0	8.0	7.0	1.0
2	Describe the role of fossils in explaining diseases and abnormalities in early man.	6.0	7.0	3.5	3.5	8.0	8.8	7.0	1.8
3	Explain the different tools used by healers to diagnose and treat diseases in early civilizations.	8.0	8.8	7.0	1.8	na	na	na	na
4	Evaluate the importance of early discoveries in medicine and pharmacy.	7.0	8.0	7.0	1.0	na	na	na	na
5	Reflect on the significance of shaman healers in prehistoric civilizations and their connection to healthcare in the contemporary era.	6.0	7.0	5.0	2.0	8.0	8.0	7.0	1.0
6	Recognize the most important contributions of the Ancient Chinese, Indians, Greeks, Romans, and Muslims to medicine and pharmacy and their impact on standardizing Western medicine and pharmacy practice today.	7.5	8.0	7.0	1.0	na	na	na	na
7	Discuss how some civilizations built upon the achievements of other civilizations in the field of medicine and pharmacy.	7.0	8.8	7.0	1.8	na	na	na	na
8	Recognize prominent individuals who contributed to the advancement of medicine and pharmacy.	8.0	8.0	7.0	1.0	na	na	na	na
9	Explain why there was a need to separate pharmacy from medicine.	7.0	8.0	5.0	3.0	7.0	7.0	7.0	0.0
10	Describe key features of early pharmacy in the Muslim civilization.	7.0	8.0	7.0	1.0	na	na	na	na
11	Discuss the achievements of Muslim scholars in the fields of medicine and pharmacy.	7.0	8.0	6.3	1.8	na	na	na	na
12	Describe the evolution of pharmaceutical industry.	7.0	8.0	7.0	1.0	na	na	na	na
13	Discuss the history of some diseases and medications used in their treatment today.	7.5	8.0	7.0	1.0	na	na	na	na

### Overview of the course, teaching, and evaluation methods

Suggestions, views, and opinions of the panelists on the proposed course, teaching, and evaluation methods were analyzed qualitatively. Number of meetings, engaging students on the different levels of Bloom’s taxonomy, different teaching and evaluation methods were suggested by the panelists.

### Number of meetings

The panelists suggested that 16 dedicated meeting hours (1 credit hour) would be sufficient to cover the course contents.

“…I think the currently dedicated 16 hours of meeting time are sufficient to cover the course.” An educator/academician with 11 years of teaching experience.

The other panelists agreed that 16 h of meeting time would be sufficient to cover the course materials.

### Using Bloom’s taxonomy

**Fig. 2:**
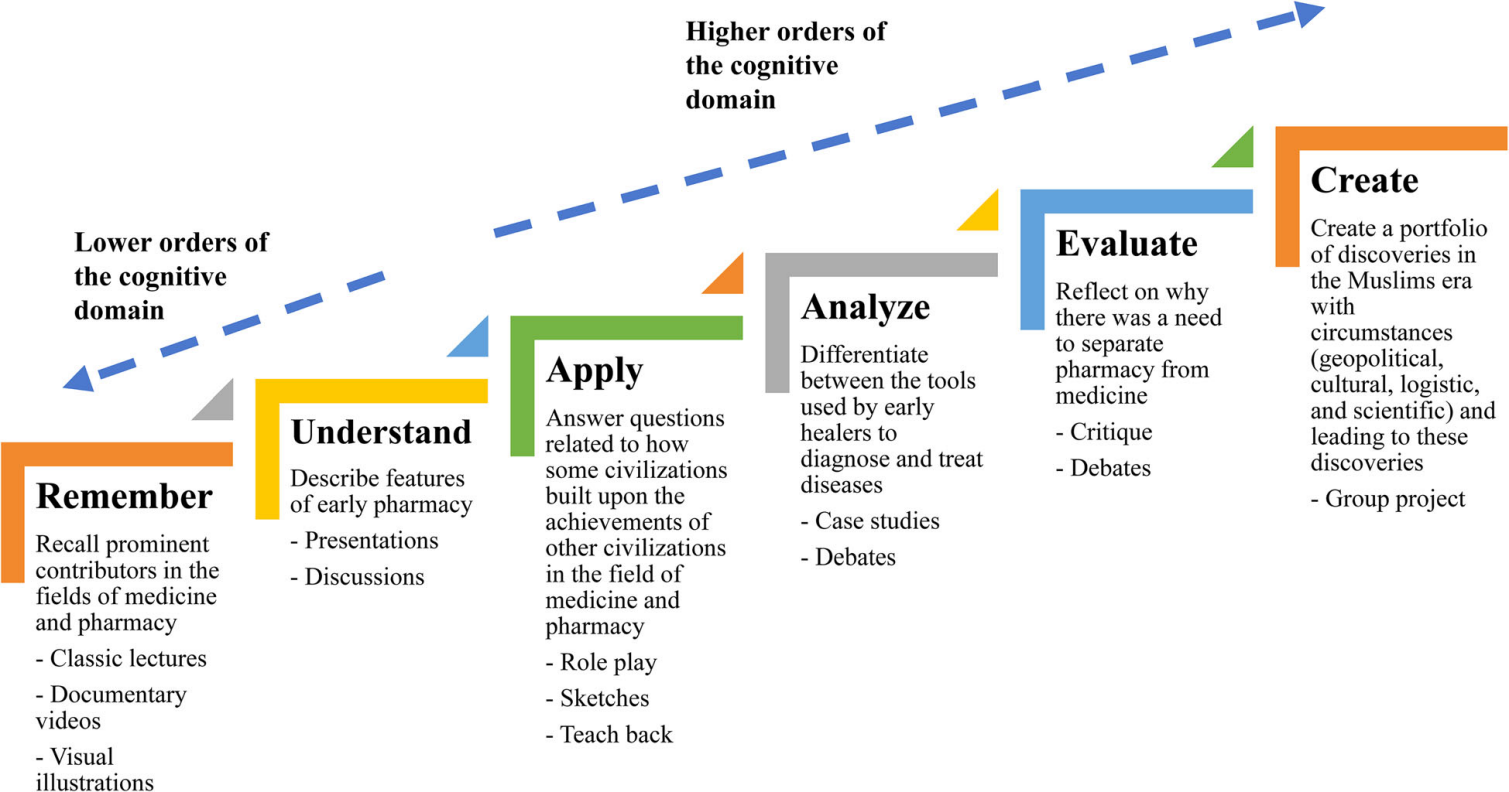
The use of Bloom’s taxonomy in targeting lower and higher orders of the cognitive domain while teaching and evaluating performance of students in the course

The panelists stressed on the importance of engaging students on the different levels of Bloom’s taxonomy. The panelists suggested the use of Bloom’s verbs like describe (knowledge) and explain (understand) to target the lower orders of the cognitive domain, and other verbs like reflect (evaluate) to target higher order of the cognitive domain. Figure 2 illustrates how Bloom’s verbs were suggested to be used to target lower and higher orders of the cognitive domain.

“In my opinion, the course should promote knowledge, curiosity, and wisdom among the students….” A physician with 9 years of practical experience.

### Teaching methods

The panelists suggested that teaching of history can be achieved largely through face-to-face lectures during which the study materials would be presented to students through PowerPoint or Preezi presentations. The panelists also suggested that oftentimes, students might be provided with reading materials to prepare in advance and engage in group discussions. Video documentaries, case studies of prominent discoveries might sometimes be presented in the class and students would be encouraged to engage in discussions and debates.

“….many students are visual learners, one can make use of the widely available video documentaries.” An educator/academician with 7 years of teaching experience.

Some panelists suggested that lectures, presentations, and group discussions might be combined to achieve the aims of the course.

“I believe students can acquire working knowledge of history to achieve some of the intended learning outcomes through lectures.” A pharmacist with 10 years of practical experience.

The panelists also suggested that students would be asked to write and submit reflections and group projects. Through discussions, debates, reflections, and group projects students are given the opportunity to apply the knowledge they receive through the lectures and presentations using the higher cognitive domain (Fig. 2).

“…it is important to target the higher orders of the cognitive domain. I think this can largely be achieved through reflections.” An educator/academician with 12 years of teaching experience.

### Evaluation methods

The panelists commented that a considerable proportion of the course requires lower order thinking skills (knowledge and understanding); therefore, multiple-choice questions (MCQs) might be used to assess students with regard to these dimensions. The panelists suggested that student portfolios of presentations, case discussions, written reflections, and group projects might also be used to evaluate the higher cognitive dimensions of the students (Fig. 2).

## Discussion

This study sought to develop consensus-based objectives and intended learning outcomes of a history of medicine and pharmacy course through the Delphi technique as a formal consensus technique. Views and opinions on 10 statements on the importance of teaching history of medicine and pharmacy to medical and pharmacy students were exposed. Additionally, consensus-based course aims (*n* = 4) and intended learning outcomes (*n* = 13) were developed. Recommendations on the course design, number of meeting hours, teaching, and evaluation methods were also exposed. This is the first study to report on the development of consensus-based objectives and intended learning outcomes of a history of medicine and pharmacy course using a formal consensus technique. Findings of this study could be valuable to decision makers in academia and educators/academicians wishing to develop courses on history of medicine and pharmacy for medical and pharmacy students in the Arab World.

Recently, there has been an increasing interest in consensus-based course contents [8, 18]. Before this study, the academic literature provided scarce guidance on objectives and intended learning outcomes of history courses for medical and pharmacy students [1, 8, 18]. For the first time, this study provided consensus-based course objectives and intended learning outcomes. Additionally, this study provided how to design a course within the limited time dedicated to this course, how to teach the course, how to engage students in activities, and how to evaluate performance of the students considering the lower and higher orders of the cognitive domain.

It has been argued that in a highly competitive environment, students as well as teaching faculty might find history a boring subject distracting students and faculty alike from concentrating more on the core courses [1, 6, 7]. In many medical and pharmacy schools, the curricula could be overwhelmingly crowded with huge amount of information that students need to learn [1, 3]. Additionally, students could barely have a moment to catch their breath amid the countless number of examinations. However, recognition of events, milestones, and individuals that contributed to the advancement of science, medicine, and pharmacy is at the heart of both professions: medicine and pharmacy because history is full of tragedies to learn from and triumphs to celebrate [1, 3, 5–7, 19–25]. Engaging students through the use of Bloom’s hierarchy and stirring their curiosities might reduce boredom reported with history courses. Findings of this study were not surprising that the vast majority of the panelists agreed on the importance of teaching history courses to medical and pharmacy students. The importance of teaching history of medicine to students was addressed in a plea by Dr. Eugene Cordell at the 105th anniversary of the Medical and Chirurgical Faculty of the state of Maryland. The statements on the importance of teaching history to medical and pharmacy students which the panelists agreed upon in this study were consistent with those stated by Dr. Cordell in his address [19].

In this study, the intended learning outcomes of the course were designed and classified in accordance with the cognitive domain of the Bloom’s taxonomy of educational objectives, which has been amended by Krathwohl and Anderson [26]. Special emphasis was placed on promoting knowledge, gratifying curiosity, broadening views, and strengthening wisdom and judgement among students [1, 3, 7, 19, 27, 28]. Because involvement of students on the different levels of Bloom’s taxonomy was required, the strategies of teaching and learning in this course were designed to be delivered accordingly. Skinner proposed his theory of behaviorist learning [29]. In response to this theory, cognitive learning theories were developed. According to these theories, students learn as a result of internal processes in which higher-order mental activities including memory, perception, thinking, problem-solving, reasoning, and concept formation take place [30]. Cognitive learning theories proposed that learning is a result of interactions between new information acquired by an individual and the existing information, specifically, when relevant structured are already existent [26, 31]. The use of Bloom’s verbs in this study might ensure targeting the lower and upper orders of the cognitive domain.

Bloom explored the concept of cognitive domain and proposed his theories relevant to this domain. Bloom’s theories and their amendments are continuously shaping the different aspects of contemporary learning and teaching [26]. Influenced by Bloom’s theories, Benner demonstrated that during professional education, learners’ transition through a developmental continuum and progress from novices to experts [20, 32]. Honey and Mumford classified learners into: activists, reflectors, theorists, and pragmatists [27, 33]. It has been argued that each of those learners could function at an educational developmental level that can directly correlated with a discreet level on the Bloom’s taxonomy [34, 35]. The only distinction between those learners could be on the way how they could arrive at their current occupancy of the level on the Bloom’s taxonomy. The course proposed in this study took into consideration the different classes of learners and how to engage them.

In this study, the panelists proposed that teaching of history could be achieved largely through face-to-face lectures during which the study materials are presented to students through a PowerPoint or Preezi presentations. Oftentimes, students would be provided with reading materials to prepare in advance and engage in group discussions. Video documentaries, case studies of prominent discoveries would sometimes be presented in the class and students would be encouraged to engage in discussions and debates [36]. These presentations, discussions, and debates would aim to: 1) facilitate engaging students in the learning process, 2) help targeting higher cognitive functions with regard to Bloom’s taxonomy, 3) train future doctors and pharmacists engage in debates on historical practice, and 4) enable cherishing memories, virtues, accomplishments, and celebrating prominent contributors.

The teaching method adopted for this course would combine lectures, presentations, and group discussions. There are many pedagogic advantages of this mixed method adopted to teaching this course [34, 35]. Lectures and presentations might enable students to acquire working knowledge of history to achieve some of the intended learning outcomes of this course. In general, lectures and presentations target the lower orders of the cognitive domain [35]. Selected documentaries would be played and students would be encouraged to engage in debates and discussions on issues brought in these documentaries. Students would also be asked to write and submit their reflections on these issues. Discussions, engaging in debates, reflections, and group projects target the higher orders of the cognitive domain [37]. Through discussions, debates, reflections, and group projects students would be given the opportunity to apply the knowledge they received through lectures and presentations using the higher cognitive domain.

A considerable proportion of the course requires lower order thinking skills (knowledge and understanding), therefore, multiple-choice questions (MCQs) would be used to assess students with regard to these dimensions [28]. It has been argued that MCQs were unable to test higher cognitive dimensions, however, MCQs have the advantage of permitting coverage of different topics and reducing grading bias and errors. Additionally, MCQs are widely used in equivalency, licensure, and board examinations [38, 39]. Student portfolios of presentations, case discussions, written reflections, and group projects would also be used to evaluate the higher cognitive dimensions of the students.

### Strengths and limitations

To interpret the findings of this study, it is important to consider a number of strengths and limitations. The strengths of this study include the following. First, this study was the first to develop consensus-based aims and intended learning outcomes of a history of medicine and pharmacy course to be taught to medical and pharmacy students. Second, the Delphi technique was used as the formal consensus method in this study. The Delphi technique is one of the widely acceptable techniques in developing consensus-based concepts on which there is a lack of consensus. Third, the panelists were diversified in terms of gender, age groups, country of origin, professions, type of employer, and length of practical experience. This diversity might have depth and width to the findings of this study. As the panelists included educators/academicians and professionals (physicians and pharmacists), the views and opinions collected in this study might reflect the views and opinions those who are involved in teaching history course and the practicing professionals (physicians and pharmacists).

However, the limitations that could be associated with this study include the following. First, this study was based on the views and opinions of the panelists who were from the Arab World. As panelists from other regions of the world were not included in this study, this would limit the applicability of the course to other regions of the world. Second, the course developed was based on the opinions of the panelists who participated in this study. Consensus-based studies are limited by design as the findings reflect the views and opinions of the participants and not necessarily the entire educators/academicians and/or professionals (physicians and pharmacists). Third, the panel size used in this study was relatively small. However, there is no consensus on the ideal size of a Delphi panel. Previous studies used panels in the sizes between 10 and 1000. The panel size used in this study was within the range of those used in previous studies. Fourth, views and opinions of the students were not included in this study. This limitation would be addressed in a separate study in which the views and opinions of the students would be collected and compared to those of the educators/academicians and those of the physicians and pharmacists.

### Implications on future practice and research

Teaching history courses to medical and pharmacy students was challenged by several issues including allocating instruction hours to teaching history, lack of lecturers/instructors specialized in history of medicine and pharmacy, lack of interest in teaching history courses to medical and pharmacy students, lack of uniformity in students who subscribed to history courses (Sophomores, Juniors, Seniors, or any who choose to attend), lack of examinations evaluating performance of students in history courses, lack of historical clubs, lack of uniformity of history courses, and lack of well-written textbooks/course packages that students can use [3, 5–7, 19].

History of medicine and pharmacy is an interesting topic to be taught to future doctors and pharmacists. Teaching and learning based on the cognitive domain in Bloom’s taxonomy of educational objectives have shown to be rigorous in terms of quality standards required in higher education. Educators should ensure that learners reach the higher levels of the cognitive domain through diverse teaching and pedagogic methods. Globally, accreditation of medical and pharmacy education has become a top priority for medical and pharmacy schools [8]. With the increasing interest in accreditation, there has been many calls to standardizing courses to meet the prerequisite global norms and standards for future practice of medicine and pharmacy [18]. Therefore, developing consensus-based courses might increase the likelihood of meeting the nationally and/or internationally accepted standards [8, 18]. The methods used in this study might inform educators/academicians in the Arab World to develop consensus-based course contents to meet the nationally and/or internationally accepted standards.

## Conclusion

Consensus-based course of history of medicine and pharmacy course was developed for medical and pharmacy students in the Arab World. Well-designed course aims, contents, intended learning outcomes, teaching, and evaluation methods are more likely to meet the accreditation requirements and might improve performance of medical and pharmacy students. Future studies are still needed to investigate if such consensus-based courses can improve performance of the students.

## Supplementary Information

**Additional file 1: Supplementary Table S1.** Adherence to Conducting and REporting of DElphi Studies (CREDES) guidelines [1].

## Data Availability

The datasets used and/or analyzed during the current study are available from the corresponding author on reasonable request.
