# Supplementary material for: Development of consensus-based aims, contents, intended learning outcomes, teaching, and evaluation methods for a history of medicine and pharmacy course for medical and pharmacy students in the Arab world: a Delphi study
Source: BMC Med Educ. 2021 Jul 16;21:386. doi: 10.1186/s12909-021-02820-7 (PMC8285807; doi:10.1186/s12909-021-02820-7)
Supplement: Supplementary file 1 — Additional file 1: Supplementary Table S1. Adherence to Conducting and REporting of DElphi Studies (CREDES) guidelines [1]. [file 12909_2021_2820_MOESM1_ESM.docx]

**Supplementary materials for the manuscript**

**Development of consensus-based** **aims, contents, intended learning outcomes, teaching, and evaluation methods for a history of medicine and pharmacy course for medical and pharmacy students in the Arab World: a Delphi study**

**Ramzi Shawahna^1,2*^**

^1^Department of Physiology, Pharmacology and Toxicology, Faculty of Medicine and Health Sciences, An-Najah National University, Nablus, Palestine

^2^An-Najah BioSciences Unit, Centre for Poisons Control, Chemical and Biological Analyses, An-Najah National University, Nablus, Palestine

* Corresponding author: Ramzi Shawahna

Ramzi Shawahna, PhD, Department of Physiology, Pharmacology and Toxicology, Faculty of Medicine & Health Sciences, New Campus, Building: 19, Office: 1340, An-Najah National University, P.O. Box 7, Nablus, Palestine

Phone: + (970) 923 45113 ext 2772

Phone: + (970) 92349739

e-mail: [ramzi_shawahna@hotmail.com](mailto:ramzi_shawahna@hotmail.com)

**Supplementary Table S1:**

Adherence to Conducting and REporting of DElphi Studies (CREDES) guidelines [[1](#_ENREF_1)]

| **#** | **Category** | **Section/paragraph in the manuscript** |
| --- | --- | --- |
|  | **Rationale for the choice of the Delphi technique** |  |
| 1 | Justification/rationale for Delphi | Background, Paragraph 3 |
| 2 | Purpose well defined | Background, Paragraph 3 |
|  | **Planning and design** |  |
| 1 | Planning and process | Methods, Planning and process |
| 2 | Selection of experts clearly justified | Methods, Selection of the panelists for the Delphi technique |
|  | **Study conduct** |  |
| 1 | Clear description of methods | Methods section. |
| 2 | Flow chart | Figure 1 |
| 3 | Informational input | Methods, Planning and process |
| 4 | Clear definition of consensus | Methods, Analysis of the votes and definition of consensus |
| 5 | Prevention of bias | Methods, The second Delphi round |
| 6 | Pilot test of instruments | Methods, Piloting and review of the questionnaire |
| 7 | Interpretation and processing of results | Methods, Analysis of the votes and definition of consensus |
| 8 | Validity | Methods, Analysis of the votes and definition of consensus |
|  | **Reporting** |  |
| 1 | Transparent reporting of results | Results section. |
| 2 | Data analysis clearly justified and reported | Results, Tables and Figures |
| 3 | Expert panel (Member of organization, recognized authority, relevant clinical academic expertise, profession/stakeholder) | Results, Table 1. |
| 4 | Information of rounds | Results, Consensus-based general aims of the course |
| 5 | Discussion of limitations | Discussion, Strengths and limitations |
| 6 | Adequacy of conclusions | Conclusion |

1. Junger S, Payne SA, Brine J, Radbruch L, Brearley SG: **Guidance on Conducting and REporting DElphi Studies (CREDES) in palliative care: Recommendations based on a methodological systematic review**. *Palliative medicine* 2017, **31**(8):684-706.
